# Silver and Copper Nanoparticles—An Alternative in Future Mastitis Treatment and Prevention?

**DOI:** 10.3390/ijms20071672

**Published:** 2019-04-03

**Authors:** Aleksandra Kalińska, Sławomir Jaworski, Mateusz Wierzbicki, Marcin Gołębiewski

**Affiliations:** 1 Cattle Breeding Division; Warsaw University of Life Sciences, Ciszewskiego 8 street, 02-786 Warsaw, Poland; aleksandra_kalinska@sggw.pl; 2 Division of Nanobiotechnology; Warsaw University of Life Sciences, Ciszewskiego 8 street, 02-786 Warsaw, Poland; slawomir_jaworski@sggw.pl (S.J.); mateusz_wierzbicki@sggw.pl (M.W.)

**Keywords:** silver, copper, nanoparticles, mastitis pathogens

## Abstract

Nowadays, mastitis is one of the biggest problems in breeding dairy cattle. Treatment of this disease with conventional antibiotics is ineffective because many pathogens are resistant. Researchers have therefore been forced to look for new solutions, and metal nanoparticles (NPs) have been found to be the most appropriate agents. This study uses commercially available silver (AgNPs) and copper (CuNPs) nanoparticles and synthetized silver–copper nanoparticles (AgCuNPs) to evaluate the effect of these NPs on human and bovine mammary cells. The effect of AgNPs, CuNPs, and AgCuNPs on pathogen species commonly involved in udder inflammation (e.g., *Staphylococcus aureus* and *Escherichia coli*) was also established. The results show that commercially available NPs were of good quality and did not have a toxic effect on mammary gland tissue. AgNPs, CuNPs, and AgCuNPs also influenced or decreased the viability of pathogens. Therefore, the presented data suggest that metal NPs could be used in mastitis prevention and treatment in the future. However, the presented preliminary results require further in vivo analysis.

## 1. Introduction

About 90% of udder inflammations (mastitis) are caused by different environmental bacteria [1]. However, references suggest that the antibiotic amounts used in the treatment of mastitis should be reduced, as inappropriate antibiotic use in the treatment of dairy cows in the past has contributed to the increased antibiotic resistance of pathogens involved in mastitis [2]. Due to this, scientists have been forced to look for new solutions in mastitis treatment. One of the most promising ideas is the possibility of using metal nanoparticles (NPs). Their antibacterial properties are of high interest due to the growing development of resistant pathogen strains [3].

Nanotechnology is a rapidly growing field in science and technology, due to the possibility of manufacturing new materials on the nanoscale level [4,5]. Nanoscale materials have a high surface area to volume ratio and unique chemical and physical properties that make them promising antimicrobial agents [6,7]. NPs are produced and utilized in a wide range of commercial products, e.g., silver nanoparticles (AgNPs) are used in electronics, bio-sensing, clothing, food production, paints, sunscreens, cosmetics, and medicine. Their common use increases human exposure to NPs and the potential risks connected with their short- and long-term toxicity [5]. AgNPs can have cytotoxic and genotoxic effects on human microvascular endothelial cells [8]. However, opinions about the toxicity of NPs vary among scientists. Different results from different studies may be connected with various factors, e.g., type or size of NPs, synthesis method, or conditions.

Some authors claim that AgNPs can be valuable antiviral agents, photosensitizers, and radiosensitizers, and can be used as anticancer therapeutic agents in, for example, leukemia, breast cancer, hepatocellular carcinoma, and lung carcinoma [9]. Recent studies suggest that AgNPs have low toxicity for mammary gland tissue and therefore should not have a negative effect on udder tissues [10]. Copper nanoparticles (CuNPs) also have high antibacterial and antifungal effects [11,12].

Nevertheless, the most important advantage of NPs is that they do not lead to bacterial resistance [13], which is currently the biggest problem in bacterial bovine mastitis treatment.

Available data shows that NPs may have a toxic effect on bacteria because of the formation of reactive oxygen species (in the Fenton reaction), DNA degradation, and lipid and protein peroxidation [14]. NPs could be an effective solution in mastitis treatment because currently, the susceptibility of bacteria to antibiotics should be diagnosed in each case [2,15] in order to determine an appropriate treatment method.

AgNPs, CuNPs, and silver–copper nanoparticles complex (AgCuNPs) are very frequently used in different studies from varied fields of science. Moreover, colloids of these NPs are widely commercially available and cost-friendly. Previously mentioned studies were focused mainly on in vitro experiments, but the results could also be used in practice, e.g., in mastitis treatment and prevention. To achieve this aim, NPs must be described as effective agents against pathogen species causing udder inflammations. Therefore, the authors of these papers decided to prepare a series of experiments to evaluate if AgNPs and CuNPs could be used in subclinical mastitis treatment and prevention, e.g., as an addition to pre-dipping and dipping disinfectant preparations. However, metallic NPs cannot be toxic for cattle mammary gland tissue and must also be safe for humans. Due to that fact, conducted studies should include the influence of NPs on cattle and human cells.

The aims of the first conducted study were: (1) To evaluate the physicochemical properties of commercially available AgNPs, CuNpS, and AgCuNPs; (2) to determine the influence of commercially available AgNPs, CuNPs, and AgCuNPs on two mammary cell lines (human and bovine); (3) to determine the influence of commercially available AgNPs, CuNPs, and AgCuNPs on selected pathogen species (*Enterococcus faecalis*, *Escherichia coli*, *Staphylococcus aureus*, *Enterobacter cloacae*, *Streptococcus agalactiae*, and yeast *Candida albicans*) that often cause mastitis.

## 2. Results

### 2.1. Physicochemical Properties of Selected Nanoparticles

The data showed that AgCuNPs occur due to the self-organization phenomenon that is a widely known fact. Zeta potential for AgNPs, CuNPs, and AgCuNPs was calculated (Table 1, Materials and Methods). Zeta potential for most of the NPs (except Ag solutions of 20 mg/L and Cu solutions of 5 mg/L) generally achieved values close to 0. Therefore, sonification is necessary in order to ensure the appropriate distribution of NPs.

The average size and distribution of selected NPs were measured using hydrodynamic light dispersion (average agglomerate size) and analysis of transmission electron microscope photographs. Single CuNPs were smaller than AgNPs. However, CuNPs have a stronger agglomeration tendency compared to AgNPs and because of that, their hydrodynamic size is higher (Table 2, Materials and methods). AgCuNPs have higher potential for agglomerate formation due to the fact that they differ in size.

### 2.2. Viability of BME-UV1 and HMEC Cells after Incubation with AgNPs, CuNPs, and AgCuNPs

The viability of BME-UV1 and HMEC cells was estimated using a PrestoBlue test (ThermoFisher, Scientific), measuring the oxidoreductive activity of cells. The test includes the resazurin-based solution permeable for cells that reduces the environment of the viable cells. Color changes can be easily observed and measured using, e.g., absorbance.

**Figure 1:**
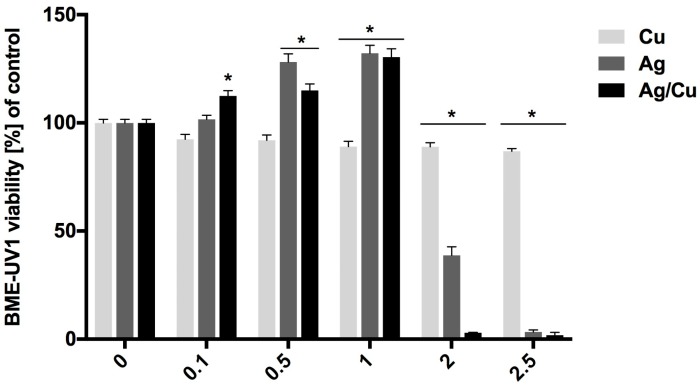
Oxidoreductive activity of BME-UV1 cells, calculated using PrestoBlue test (*—differences significant at *p* ≤ 0.05).

The oxidoreductive activity of BME-UV1 cells increased after incubation in the mediums containing 0.5 mg/mL and 1 mg/mL of AgNPs and the mediums containing 0.1, 0.5, and 1 mg/L of AgCuNPs. Higher concentrations of AgNPs and AgCuNPs decreased the oxidoreductive activity of BME-UV1 cells. In the mediums containing 2 and 2.5 mg/L of CuNPs, cell viability was decreased. The obtained results are presented in Figure 1.

**Figure 2:**
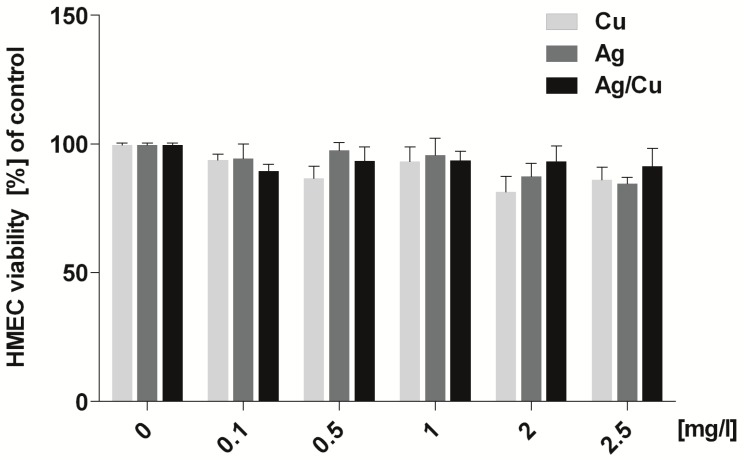
Oxidoreductive activity of HMEC cells, calculated using PrestoBlue test (no statystical differences).

Data regarding the viability of HMEC cells after incubation in mediums containing AgNPs, CuNPs, or AgCuNPs at concentrations of 0.1, 0.5, 1, 2, and 2.5 mg/L are presented in Figure 2. The concentrations of NPs did not decrease the oxidoreductive activity of HMEC cells and therefore did not have a cytotoxic effect.

### 2.3. Membrane Integrity of BME-UV1 and HMEC Cells after Incubation with AgNPs, CuNPs, and AgCuNPs

**Figure 3:**
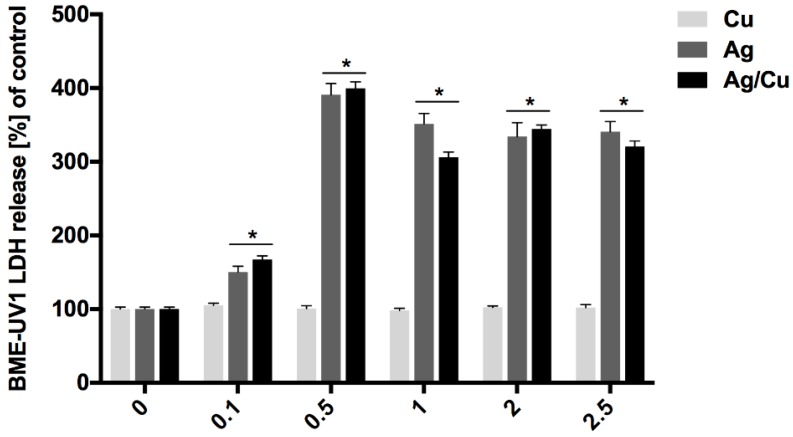
Membrane integrity of BME-UV1 cells (*—differences significant at *p* < 0.05).

**Figure 4:**
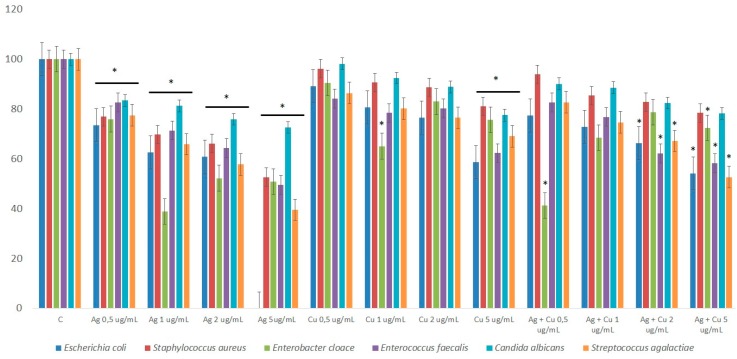
Membrane integrity of HMEC cells (*—differences significant at *p* < 0.05).

The membrane integrity of BME-UV1 and HMEC cells was evaluated after incubation in mediums containing AgNPs, CuNPs, or AgCuNPs at concentrations of 0.1, 0.5, 1, 2, and 2.5 mg/L, using an LDH Cytotoxicity Assay Kit (ThermoFisher Scientific, Waltham, MA, USA). Lactate dehydrogenase (LDH) is a cytosolic enzyme that can be used as the indicator of cellular toxicity and provides a safe alternative to radioactive cytotoxicity assays because it is a reliable colorimetric test that quantitatively measures the amount of LDH released into the media from damaged cells. Obtained data are presented in Figure 3. CuNPs did not have an impact on cell membrane integrity. Incubation in AgNP and AgCuNP mediums resulted in an elevated level of LDH. The results of membrane integrity of HMEC cells are presented in Figure 4. Solutions with concentrations of 0.5, 1, 2, and 2.5 mg/L of AgNPs, AgCuNPs, and CuNPs influenced a spontaneous leak of LDH by 20–60% and increased cell membrane integrity (*p* < 0.05).

### 2.4. Bacterial and Fungi Cultures

**Figure 5:**
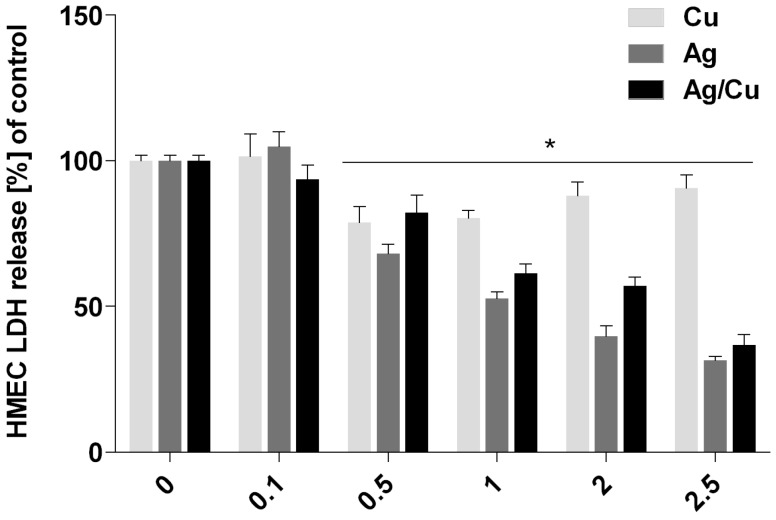
Viability of selected pathogen species after incubation in solutions with different concentrations of nanoparticles (NPs) (*—differences significant at *p* < 0.05).

The viability of pathogen cells was expressed as a percentage of the control group and presented in Figure 5. The number of cells in control group (C) was presented as 100% and compared with the number of cells in the experimental groups. Pathogen viability decreased in all experimental groups with NPs. The addition of AgNPs resulted in the highest decrease in the number of pathogen cells and differences were significant at *p* < 0.05.

The presented results will be used in further experiments. The aim of the next experiment will be to estimate the influence of popular cosmetic substrates on the pathogens’ viability and prepare initial mixtures containing commercially available cosmetic substrates with the addition of NPs. These mixtures should care for the udder and teat skin (e.g., protect and moisturize), in addition to having antibacterial properties. Therefore, the biggest challenge will be choosing the most effective NP concentration and composition.

## 3. Discussion

### 3.1. Zeta Potential and NP Size

Nanotechnology is currently a subject of interest for researchers from different fields of science. Gold NPs have been described as the most stable metal NPs [16]. Furthermore, some authors suggest their positive influence and possible use in medicine, e.g., for cancer treatment [17] or as support in radiation therapy [18]. Platinum NPs can also be involved in radiation therapy or cancer treatment [19]. The highest reactivity of NPs is observed when NPs are placed in a new environment and are not covered by other substances or structures, such as bacteria, proteins, or lipids [20]. Studies focusing on the synthesis and properties of NPs have been presented in several papers [11,21,22,23,24,25]. However, the properties of metal NPs still need further analysis if we want to use metallic NPs in mastitis prevention in dairy cattle.

Zeta potential and dynamic light scattering (DLS) are often used to evaluate the properties of NPs. Zeta potential is directly proportional to the antibacterial activity of AgNPs [26]. Our analysis revealed that zeta potentials for AgNPs, CuNPs, and AgCuNPs at concentrations of 20 mg/L were −26.30 mV, −9.25 mV, and −1.13 mV, respectively. Similar results for AgNPs were obtained in studies presented by, e.g., Sadowski et al. [27], Oukarroum et al. [28], and Ramalingmam et al. [24]. On the other hand, Zain et al. [23] showed that zeta potential for AgNPs, CuNPs, and AgCuNPs reached values of 27.8 to 33.8 mV. A previous paper revealed that AgNPs are stable with a zeta potential more positive than +30 mV or more negative than −30 mV [29]. However, zeta potential for AgCuNPs was +35.2 mV in a different paper [23]. These differences could be due to the methods of synthesis and used substrates. Zeta potential can be one of several factors influencing the agglomeration tendency of NPs. Therefore, it would probably be more effective to standardize the size of nanoparticles if we plan to use NPs in disinfectant preparations.

In this study, the average size of single AgNPs varied from 35 to 100 nm, for CuNPs, from 0.5 to 80 nm, and for AgCuNPs, achieved 5–100 nm. The varied results of synthesized NPs can be the result of the quality of commercially available NP colloids, human mistakes during the synthesis, and the conditions of this process, according to AgCuNPs. Studies carried out by Paszkiewicz et al. [30] suggest that the methods of NP synthesis influence the average size of NPs. In their studies, the average size of AgNPs was 40 nm using NaBH4, and was even 10 times lower in comparison to NPs received using N_2_H_4_ reducing agent [30]. A similar phenomenon was also observed for CuNPs in their studies. Zain et al. [23] reported that the concentration of substrates used in synthesizing NPs is important, and the size of NPs increased almost proportionally to substrate concentration. Previous studies also revealed that synthesis methods affect the size of NPs, e.g., AgNPs were slightly larger than CuNPs in studies carried out by Zain et al. [23]. Ramalingmam et al. [24] reported that the average size of the synthesized AgNPs was 64.30 nm. The average diameter of monodispersed CuNPs studied by Kruk et al. [11] was 50 nm.

The average size of AgCuNPs varied from 5 to 100 nm in this study. The shape of obtained NPs was spherical, which is a well-known fact, and was found, e.g., in studies by Paszkiewicz et al. [30]. However, the differences in the size of AgCuNPs in their paper were smaller, as the average size was 45–50 nm.

### 3.2. Viability and Integrity of BME-UV1 and HMEC Cells

The cytotoxicity of metal NPs against different cell lines has been the subject of interest in many research studies, and results vary according to the field of science. Nevertheless, papers analyzing the influence of AgNPs, CuNPs, and AgCuNPs on BME-UV1 and HMEC cells are limited. The toxicity of NPs is a crucial issue for both cattle and human health and safety, and must be carefully studied.

Data differ among scientists. Some authors have demonstrated their lack of toxicity, e.g., for human cells [31], while others argue that NPs can be toxic and negatively influence living cells [5]. Studies focusing on the cytotoxicity of NPs against bovine mammary epithelial cells suggest that NPs do not have a toxic effect on udder tissue [10].

In our study, the oxidoreductive activity of BME-UV1 cells increased after incubation in mediums containing 0.5 and 1 mg/mL of AgNPs and in mediums containing 0.1, 0.5, and 1 mg/L of AgCuNPs. However, higher concentrations of selected NPs decreased the oxidoreductive activity of BME-UV1 cells. On the other hand, our data on HMEC cell viability after incubation in mediums containing AgNPs, CuNPs, or AgCuNPs at concentrations of 0.1, 0.5, 1, 2, and 2.5 mg/L did not decrease the oxidoreductive activity of HMEC cells and therefore did not have any cytotoxic effect. These results can suggest that too high concentrations of NPs can have a negative impact on mammary gland cells. The available references focused on water animals often present similar data. The higher concentrations of NPs are generally toxic, e.g., for larvae development and animal organs [32,33]. At the same time, the observed differences in the oxidoreductive activity of used cell lines can be the result of errors during pipetting or passaging the cells.

LDH is a cytoplasmic enzyme occurring in all tissues and is commonly used to assay cell membrane integrity. The elevation of LDH is associated with different causes of cell damage [34,35]. In this study, CuNPs did not have an impact on the membrane activity of BME-UV1 cells, but incubation with AgNPs and AgCuNPs resulted in an elevated level of LDH. Solutions with concentrations of 0.5, 1, 2, and 2.5 mg/L of AgNPs, AgCuNPs, and CuNPs influenced the spontaneous leak of LDH by 20–60% and increased the membrane integrity of HMEC cells (*p* < 0.05). Recent studies have shown that many cellular proteins can adhere to AgNPs and form the protein corona. Therefore, the LDH assay, treated as a conventional cell viability test, should be carefully used for the assessment of AgNPs [36].

### 3.3. Antibacterial and Antifungal Effect of NPs

Available references suggest that the combination of AgCuNPs has a strong antimicrobial effect [23,24]. AgNPs and CuNPs should be the most effective solution in mastitis management due to their synergistic effect on various pathogens. Studies focusing on AgNPs and CuNPs in mastitis treatment suggest that metal NPs can be an effective solution [37]. Previous studies have shown that AgNPs can also be used in diseases caused by algae [10] that are also involved in udder inflammations. There are also papers suggesting that CuNPs display antifungal activity against the *Candida* species [11], which can cause mastitis. It has also been reported that CuNPs demonstrate a significant inhibitory activity against several bacteria species, e.g., *Escherichia coli*, *Klebsiella pneumoniae*, *Pseudomonas aeruginosa*, *Propionibacterium acnes*, and *Salmonella typhi* [12].

Some researchers have suggested that single NPs can present a stronger effect on pathogens, e.g., Zain et al. [23] reported that AgNPs displayed higher activity than CuNPs and AgCuNPs. The authors also suggested that CuNPs, in an equal concentration to AgNPs and a mixture of NPs, achieved more lethal influence on the bacteria due to a higher surface area. The antibacterial activity of AgCuNPs was the highest, with minimum inhibitory concentrations of 0.054 and 0.076 mg/L against *Bacillus subtilis* and *E. coli*, respectively [23]. NPs may also have a toxic effect on bacteria because of the formation of reactive oxygen species (in Fenton reaction), the peroxidation of lipids and proteins, and the degradation of DNA [14].

In our experiment, NPs decreased *Enterococcus faecalis*, *Escherichia coli*, *Staphylococcus aureus*, *Enterobacter cloace*, *Streptococcus agalactiae*, and yeast *Candida albicans* in each group. These results confirmed previous studies in which AgNPs and CuNPs showed strong antibacterial effects.

AgNPs displayed the strongest antibacterial effect and the viability of pathogens was the lowest (*p* < 0.01). Similar results were presented in previous studies for synthesized AgNPs that displayed inhibitory activity against Gram-negative and Gram-positive bacteria like *E. coli*, *Pseudomonas aeruginosa*, *Salmonella paratyphi*, *Bacillus subtillis*, *Staphylococcus aureus*, and *Bacillus cereus* [24]. Gram-positive bacteria like *S. aureus* have a thick peptidoglycan layer in their wall that makes them less susceptible to the toxicity of AgNPs, in comparison to Gram-negative bacteria [38]. CuNPs are more toxic for Gram-positive bacteria [39] because of the high amount of amine and carboxyl groups that are part of cell membranes in Gram-positive bacteria [40]. CuNPs also displayed high activity against Gram-positive bacteria, including methicillin-resistant *S. aureus*, which was comparable to the activity displayed by AgNPs and some antibiotics [11]. However, our data did not confirm that CuNPs showed higher toxicity against Gram-positive bacteria.

According to Wang et al. [21], metallic and ionic forms of copper produced hydroxyl radicals that caused damage to proteins and DNA. However, Pramanik et al. [22] suggested that toxicity of CuNPs depends on several factors (e.g., temperature, pH, bacteria concentration, aeration). The authors pointed out that higher temperature, better aeration, and low pH conditions improve the toxicity of CuNPs because the decreased agglomeration results in more surface area for interaction with the membranes of bacterial cells. Similar conclusions were presented in studies carried out by Hajipour et al. [41], who recommended that other scientists change the construction of future experiments by focusing on specific bacteria of NPs rather than randomly chosen ones [41]. Moreover, the authors stated that two main factors are connected with the antibacterial activity of NPs: The physicochemical properties of NPs and the species of bacteria.

Another interesting possibility in bacterial bovine mastitis treatment could be the potential of synergistic effects of biogenic NPs with antibiotics, such as erythromycin, chloramphenicol, ampicillin, and kanamycin [42]. For example, ampicillin damages cell membranes and mediates the internalization of AgNPs into bacterial cells [42].

## 4. Materials and Methods

### 4.1. Preparation of AgNPs Based on Self-Organization Phenomenon

AgCuNPs were prepared by mixing AgNP hydrocolloid at a concentration of 50 mg/L (Nano-Tech, Warsaw, Poland) and a CuNP hydrocolloid at a concentration of 50 mg/L (Nano-Tech), using a 1:1 ratio. Solutions of AgCuNPs were incubated for 24 h at a temperature of 24 °C.

### 4.2. Physicochemical Properties of Selected NPs

**Figure 6:**
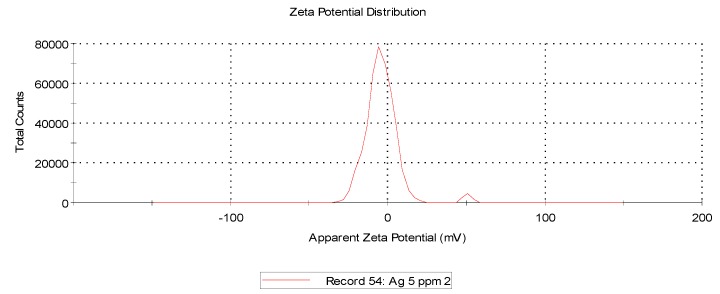
Representative distribution of zeta potential of AgNPs at a concentration of 5 mg/L.

**Figure 7:**
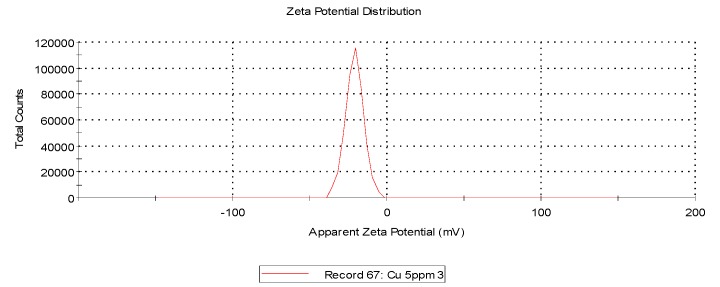
Representative distribution of zeta potential of CuNPs at a concentration of 5 mg/L.

**Figure 8:**
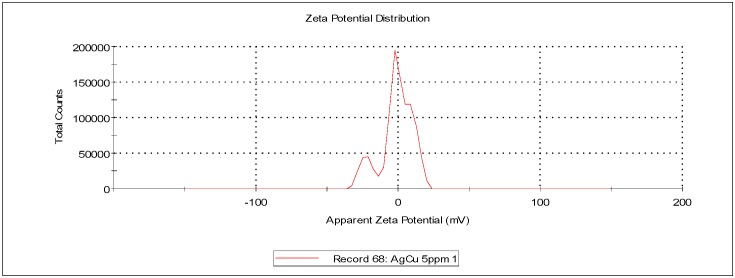
Representative distribution of zeta potential of AgCuNPs at a concentration of 2.5 + 2.5 mg/L, respectively.

Zeta potential was calculated using Zetasizer Nano ZS (ZEN3500, Malvern Instruments, Malvern, UK). The zeta potential, mobility, and conductivity of AgNPs and CuNPs at concentrations of 5 and 20 mg/L were defined. The zeta potential, mobility, and conductivity of AgCuNP complexes at concentrations of 2.5 and 10 mg/L were also obtained. The zeta potential values of selected NPs are presented in Table 1 and representative repetitions of zeta potential distribution and mobility are presented in Figure 6, Figure 7 and Figure 8.

The average size of selected NPs was measured using transmission electron microscopy and hydrodynamic size (average agglomerate size). Dynamic light scattering (DLS), used to estimate hydrodynamic size measurements, was conducted using hydrocolloids of 2.5 mg/L concentrations (for AgCuNPs, the concentration of both Ag and Cu was 1.25 mg/L). The obtained results are presented in Table 2.

**Figure 9:**
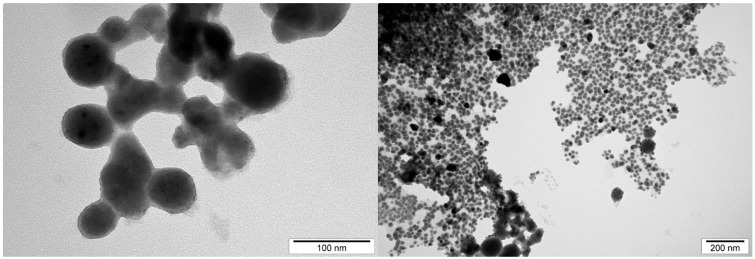
Transmission electron microscope photographs of AgNPs.

**Figure 10:**
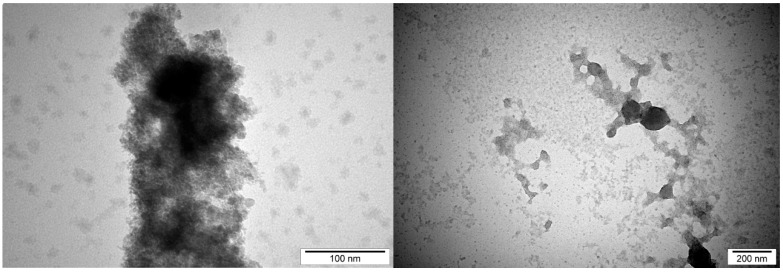
Transmission electron microscope photographs of CuNPs.

**Figure 11:**
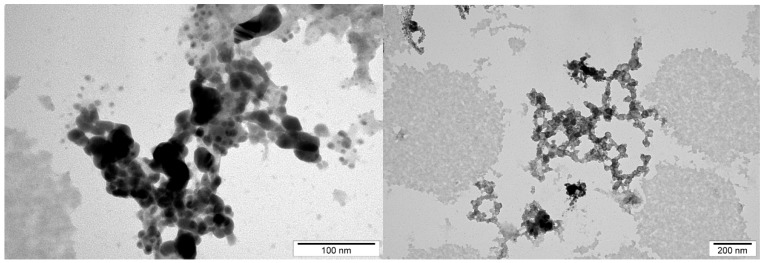
Transmission electron microscope photographs of AgCuNPs.

Transmission electron microscopy was used to present the structure of AgNPs, CuNPs, and AgCuNPs. Pictures of selected NPs are presented in Figure 9, Figure 10 and Figure 11.

### 4.3. BME-UV1 and HMEC Cells Cultures

Bovine mammary gland cells from the BME-UV1 line were provided by M. Gajewska (Veterinary Medicine Faculty, Warsaw University of Life Sciences). BME-UV1 cells were kept in a DMEM/F12 medium (ThermoFisher Scientific), supplemented with 10% bovine serum and additions of antibiotic and antimycotic agents (ThermoFisher Scientific). Cells were cultured in bottles for adherent cell culture. BME-UV1 cells were cultured at a temperature of 37 °C and in an atmosphere with 5% CO_2_ in a NuAir DH AutoFlow CO2 incubator (Polymouth, MN, USA).

HMEC cells were cultured in an HMEC-ready medium (ThermoFisher Scientific), containing an extract of the bovine pituitary gland (BPE) at a concentration of 50 μg/mL (ThermoFisher Scientific), with a HuMEC Supplement Kit (ThermoFisher Scientific) and antibiotic and antimycotic agents (ThermoFisher Scientific). Cells were cultured in bottles for adherent cell culture. HMEC cells were cultured at a temperature of 37 °C and an atmosphere with 5% CO2 in a NuAir DH AutoFlow CO_2_ incubator (Polymouth, MN, USA).

### 4.4. Viability of BME-UV1 and HMEC Cells after Incubation with AgNPs, CuNPs, and AgCuNps

The NP solutions were prepared according to the method described in this paper.

The viability of the BME-UV1 and HMEC cells was calculated using a PrestoBlue test (ThermoFisher Scientific). Presto Blue reagent is a ready-to-use resazurin-based solution, which is permeable for cells. This is a cell viability indicator that uses the reducing power of living cells. The reagent is modified by reducing the environment of the viable cells and turns from blue to pink in color, becoming highly fluorescent. Therefore, color changes can be estimated using absorbance or fluorescence measurements. BME-UV1 and HMEC cells were placed in 96-well plates in the amount of 5 × 10^3^ cells per well and were incubated for 24 h. In the next step, the medium was removed and 90 μL of medium with colloids of AgNPs, CuNPs, or AgCuNPs at concentrations of 0.1, 0.5, 1, 2, and 2.5 mg/L were added per well. The control group consisted of cells kept in a medium without the addition of NPs. After 24 h of incubation, 10 μL of PrestoBlue reagent was added to each well. Prepared plates were incubated for 2 h at 37 °C. Absorbance was measured using a wavelength of 570 nm in an immunoenzymatic Infinite M200 reader (Tecan, Durham, NC, USA).

### 4.5. Membrane Integrity of BME-UV1 and HMEC Cells after Incubation with AgNPs, CuNPs, and AgCuNPs

The amount of lactate dehydrogenase (LDH) was used to assess the membrane integrity of BME-UV1 and HMEC cells. BME-UV1 and HMEC cells were placed in a 96-well plate in the amount of 5 × 10^3^ cells per well and were incubated for 24 h. In the next step, the medium was removed and 90 μL of medium with colloids of AgNPs, CuNPs, or AgCuNPs at concentrations of 0.1, 0.5, 1, 2, and 2.5 mg/L were added per well. The control group consisted of cells kept in a medium without the addition of NPs. After 24 h of incubation, plates were centrifuged and 50 μL of medium was placed in new 96-well plates. An LDH working solution was added to each well, according to the producer’s instructions (LDH Cytotoxicity Assay Kit, ThermoFisher Scientific). The assay quantitatively measures extracellular LDH release in culture media, due to an enzymatic reaction. It results in a red formazan product that can be measured spectrophotometrically. Plates were incubated for 20 min at room temperature without light. In the next step, the reaction was completed by adding 50 μL of stop solution. Absorbance was measured using a wavelength of 490 nm in an immunoenzymatic Infinite M200 reader (Tecan).

### 4.6. Statistical Analysis

The obtained data were analyzed using one-way analysis of variance (ANOVA) in the STATGRAPHICS^®^ Centurion 17.2.05 program (StatPoint Technologies, Inc., Warrenton, VA, USA). Differences between groups were estimated using Duncan’s test. Results were presented as average values with standard deviations. Differences at *p* ≤ 0.05 were considered statistically significant.

### 4.7. Bacterial and Fungi Cultures

The bacteria of several species, *Enterococcus faecalis*, *Escherichia coli*, *Staphylococcus aureus*, *Enterobacter cloace*, *Streptococcus agalactiae*, and yeasts *Candida albicans*, were obtained from LGC Standards (Łomianki, Polska). Bacteria were kept in a 20% glycerol solution at −20 °C.

Bacterial cells were thawed and rinsed with sterile distilled water in order to remove glycerol. In the next step, bacterial cells or fungi were added to a nutrient broth medium (Bio-Rad, Warsaw, Poland) that was sterilized in glass flasks in an autoclave (Classic 2100, Prestige Medical, Chesterfield, UK). Flasks were placed in a rotating incubator at a temperature of 37 °C (SI500).

### 4.8. Preliminary NP Concentrations

Bacterial cells pipetted from the cell culture and incubated for one night (at 37 °C) were used. Experimental groups contained nutrient broth and AgNPs, CuNPs, or AgCuNPs at concentrations of 0.5, 1, and 2.5 µg/mL. The control group was nutrient broth without the addition of NPs. Each group was prepared in three repetitions. In the next step, 100 μL of the microorganism species mentioned before were added to prepared flasks. Samples were incubated for 24 h in a rotating incubator (SI500) at a temperature of 37 °C with a rotation speed of 70 rounds per minute. The viability of microorganisms was calculated using a PrestoBlue test. After the incubation, 90 µL of medium was placed in 96-well plates and 10 µL of PrestoBlue reagent (ThermoFisher Scientific) was added to each well. Each sample was placed in the plate in six repetitions. Plates were incubated for 20 min at 37 °C. Absorbance was measured using a wavelength of 570 nm in an immunoenzymatic Infinite M200 reader (Tecan). The viability of pathogens was presented as a percentage of the viability of the control group, according to the following equation:

X = (optical sample density × 100%)/optical control group density

X = pathogen viability

## 5. Conclusions

In conclusion, the NPs analyzed in this study did not reveal a toxic effect on BME-UV1 or HMEC cells, but an elevated LDH level was observed after the incubation of NPs with BME-UV1 cells. Therefore, the addition of NPs should be safe for cattle and humans. The results suggest that NPs should not have a negative effect for bovine and human cells, especially in lower concentrations. Therefore, disinfectant preparations with the addition of NPs should be safe for cows’ and humans’ health. All selected pathogen species had decreased viability in comparison to the control group, but AgNPs showed the strongest antimicrobial effect (*p* < 0.01). However, using AgNPs and CuNPs in further analysis could be the best solution because many references suggest that their toxic influence on bacteria viability varies, according to pathogen species. Another important issue is the unification of the average size of NPs to avoid agglomeration. The results obtained in this study will be used to prepare disinfectants that could be used in the milking routine of dairy cows.

## Figures and Tables

**Table 1:** Physicochemical properties of silver (AgNPs), copper (CuNPs), and synthetized silver–copper nanoparticles (AgCuNPs).

Group	Concentration	Zeta Potential	Mobility	Conductivity
	mg/L	mV	µmcm/Vs	mS/cm
AgNPs	5	−3.73	−0.2925	0.0326
AgNPs	20	−26.30	−2.0613	0.0176
CuNPs	5	−20.93	−1.6393	0.0118
CuNPs	20	−9.25	−0.7241	0.0166
AgCuNPs	2.5 Ag + 2.5 Cu	−1.07	−0.0838	0.0312
AgCuNPs	10 Ag +10 Cu	−1.13	−0.0887	0.0252

**Table 2:** Distribution of size and structure of selected NPs.

	Hydrodynamic Size (Peak 1/Peak 2) (nm)	NP Diameter	NP Structure
		(nm)	
Measurement Method	Dynamic Light Scattering (DLS)	Transmission Electron Microscopy	Transmission Electron Microscopy
Defined Parameter	Average Size of Agglomerate	Size of Single NP	NPs Form
AgNPs	262 (130.7/1571)	35–100	spherical
CuNPs	288.6 (0.6883/154.5)	0.5–80	spherical
AgCuNPs	356.2 (184.8/ 1157)	5–100	spherical
